# Coupling of high-resolution mass spectrometer and photosynthesis system for comprehensive leaf volatile metabolite profiling

**DOI:** 10.1186/s13007-026-01531-8

**Published:** 2026-05-22

**Authors:** Kelsey R. Carter, Christian Mark Salvador, Savana Colegate, Alyssa Carrell, Jun Hyung Lee, Robert Smith, Marshal McDonnell, Sara Jawdy, David McLennen, Tyler Hackworth, Lianhong Gu, Melanie A. Mayes, Udaya Kalluri, Thomas D. Sharkey, David J. Weston

**Affiliations:** 1 Environmental Sciences Division, Oak Ridge National Laboratory, Oak Ridge, TN USA; 2 Biological Sciences Division, Oak Ridge National Laboratory, Oak Ridge, TN USA; 3 Department of Environmental Biology, State University of New York College of Environmental Science and Forestry, Syracuse, NY USA; 4 Computer Science and Mathematics Division, Oak Ridge National Laboratory, Oak Ridge, TN USA; 5 Department of Botany, University of Wisconsin-Madison, Madison, WI 53706 USA

**Keywords:** Biogenic volatile organic compounds, Tandem instrumentation, PTR-ToF-MS, Volatolomics, Photosynthesis

## Abstract

**Background:**

Leaf-level biogenic volatile organic compounds (BVOCs) emissions represent a major source of organic gases in the atmosphere, influencing both climate and air quality. These emissions are strongly driven by environmental perturbations, which affect individual plant- to ecosystem-level processes. Uncovering all the BVOCs and understanding how their emissions respond to altered environmental conditions provide critical insights into vegetation-driven changes in atmospheric chemistry. We developed a tandem instrumentation setup that integrates a proton transfer reaction time-of-flight mass spectrometer (PTR-ToF-MS) with parts-per-trillion detection limits and a photosynthetic infrared gas exchange system for the untargeted survey of all the BVOCs. This novel system enables simultaneous, real-time monitoring of BVOC emissions and photosynthetic parameters at the leaf level, offering new opportunities to disentangle the physiological and environmental drivers of VOC release. Furthermore, we established the VOC Analysis and Processing Optimization Resource (VAPOR), an open-access software tool designed for rapid data post-processing and the analysis of the variability of hundreds of BVOCs. We assessed the performance of the tandem system under varying background conditions, using standard gas mixtures and a range of environmental factors.

**Results:**

Blank emissions were substantially lower for major BVOCs (e.g., isoprene) compared to those observed in plant emissions. Despite this, the observation of background-level VOCs highlights the importance of routinely acquiring and accounting for blank measurements in analyses using the coupled instrumentation. Introduction of known VOC concentrations to the system demonstrated a linear response across different compounds with varying molecular compositions, indicating minimal gas loss regardless of chemical moieties within the coupled instrumentation. We applied the optimized system to investigate the physiological mechanisms driving BVOC emissions across different genotypes of poplar and pennycress. The high mass resolution capabilities of the PTR-ToF-MS, coupled with comprehensive VAPOR-driven data analysis, enabled the identification of several important BVOCs, including methanol and methanethiol; these BVOCs displayed substantial variation across pennycress genotypes and showed concentrations ~ 100–350% higher than the blank. Moreover, isoprene emissions varied significantly among poplar genotypes grown in different potting media.

**Conclusions:**

Tandem instrumentation offers a powerful tool for profiling volatile molecular markers and elucidating their genetic and environmental underpinnings. This approach enhances our ability to predict BVOC emissions in response to genotype by environmental interactions and contributes to a deeper understanding of vegetation responses to environmental changes.

**Supplementary Information:**

The online version contains supplementary material available at 10.1186/s13007-026-01531-8.

## Background

Volatile organic compounds (VOCs) are important atmospheric chemical components that are emitted from different sources and subsequently react with oxidants such as hydroxyl radicals (OH), ozone (O_3_), and nitrate (NO_3_) and can then form additional ozone and oxidized molecules [1–4]. In natural ecosystems, terrestrial vegetation and marine organisms emit biogenic VOCs (BVOCs) that are estimated to have 10 times higher atmospheric concentrations compared to those of the organic compounds generated from anthropogenic activities such as transportation and biomass burning [5, 6]. Vegetative BVOC emissions often occur in response to biotic and abiotic factors, such as environmental stressors (e.g., heat), plant-to-plant communication, attraction of pollinators, and defense against insects and other predators [7–11]. The most prominent BVOCs are isoprene (C_5_H_8_) and monoterpenes (C_10_H_16_) [5], which include many species such as α-pinene, β-pinene, limonene, δ-carene, ocimene, and sabinene. These monoterpene species act as key contributors to the generation of low volatility organic compounds through secondary atmospheric aerosol formation. Isoprene is generated during the light-dependent metabolism in the chloroplast through the 2-C-methyl-D-erythritol 4-phosphate pathway [12]. This BVOC has a global estimate between 210 and 990 Tg C yr^− 1^, making its emissions comparable with those of methane [5].These compounds condense irreversibly to existing aerosol nuclei [13–15], which later impact cloud formation and ultimately global climate [16]. Understanding the emission of BVOCs, including both major and minor compounds from vegetation, is critical in understanding the influence of natural emissions on radiative forcing.

Within the plant, the leaf is the primary organ producing BVOCs as it is the primary site of metabolic processes, such as photosynthesis and respiration, where many VOCs are produced and released. These metabolic processes are typically investigated using leaf-level gas exchange measurements, which provide estimates of the biochemical processes controlling photosynthesis. In situ measurements are key, as any minor changes in environmental conditions alter photosynthetic rates. Parameters collected at the leaf level can estimate molecular function, and assessments of these mechanisms allow us to scale processes for use in terrestrial biosphere models [17, 18]. Emissions of several key VOCs identified as important for plant stress tolerance are directly related to the products of the photosynthetic Calvin–Benson Cycle. VOC formation is energetically expensive and directly dependent on availability of the sugar compound produced by the Calvin Cycle, glyceraldehyde 3-phosphate (GAP) and pyruvate for the chloroplastic methylerythritol 4-phosphate (MEP pathway) [19], whose products are used to produce both monoterpenes and isoprene [20, 21]. Under heat stress, some plants enhance the production of these BVOCs, which can trigger cascades of plant defense responses and allow maintained photosynthetic rates at higher temperatures [21–23]. Pairing leaf-level gas exchange with direct measurements of VOC emissions allows tightly coupled understanding between photosynthesis and production of these beneficial but costly compounds.

Leaf-level physiology and BVOC emission analyses previously involved portable photosynthesis systems (PPSs) and a fast isoprene sensor, respectively [24, 25]. Using such tandem instrumentation, the suppression of isoprene emission by CO_2_ was found to be independent of photosynthesis and can be counteracted by high temperature [26]. The integration of CO_2_/water analyzer with a fast isoprene analyzer revealed the impact of temperature on the massive release of isoprene postillumination [27]. Chemical ionization mass spectrometers (CIMS) were also paired with PPS to investigate environmental perturbations on VOC emissions. Coupled PPS and CIMS in negative mode ionization was used to evaluate the variation of spearmint leaf-level emission of formic acid in response to temperature changes [28]. Positive mode ionization (i.e., proton-transfer reaction) CIMS was also connected to PPS to investigate the impact of leaf wounding and darkening on the emission of methanol, hexanal, C6 aldehydes, alcohols, isoprene, and other green leaf volatiles of *Dactylis glomerata* [29]. A custom-built temperature-controlled and fan-stirred glass cuvette was also paired to a proton-transfer-reaction mass spectrometry (PTR-MS) instrument to continuously monitor the emission of 2-methyl-3-buten-2-ol (MBO) and total monoterpenes from ponderosa pine [30]. The tandem instrumentation showed that MBO dominated the total VOC emission, with primary control by light and temperature. A Walz GFS-3000 leaf cuvette was connected to a proton-transfer-reaction time-of-flight mass spectrometer (PTR-ToF-MS) 8000 to monitor BVOC emissions from leaf wounding of five tropical agricultural species [31].

The pairing of the PPS with rapid detection of VOC is critical for leaf-level analysis, particularly for the investigation of the physiological mechanism regulating the emission of BVOCs. However, most of these previous works that pair these systems focused on the major BVOCs [29], with minimal consideration for untargeted analysis of the whole VOC mass spectra. Besides the well-studied major plant BVOCs such as isoprene and monoterpenes, leaves also emit a wide range of lesser-known VOCs at lower concentrations. Many of these compounds remain poorly characterized or unidentified due to the low concentration of the unexplored BVOCs (i.e., parts per trillion level), the complexity of plant VOC mixtures, and the diversity of plant species. Here, we present the development, characterization, and application of the tandem of LI-6800 (PPS) with a PTR-ToF-MS 6000 × 2, a coupling that can simultaneously capture the real-time variability of wide range of unexplored VOCs and monitor the physiological activities of vegetation during the leaf-level emissions of BVOCs. The response of the system to varying known concentrations of BVOCs with different molecular backbones was also tested. The optimized instrument pairing was then applied in the detection of real vegetation (i.e., pennycress and poplars) emission with different genotypes and soil media using direct measurement and offline collection procedures (i.e., canister). This work distinguishes itself from prior studies on tandem coupling of photosynthetic systems with PTR-ToF-MS [29–33] by conducting a comprehensive evaluation of instrumental performance, enabling a broader untargeted profiling of minor biogenic volatile organic compounds (BVOCs), and integrating a high-throughput algorithm for automated data processing and analysis.

## Methods

### Description of instrumentation and connection procedures

The tandem instrumentation used in this study consisted of a PTR-ToF-MS 6000 × 2 (Ionicon Analytik Ges.m.b.H., Innsbruck, Austria) and the portable photosynthesis system (LI-6800) with a Multiphase Flash™ Fluorometer (LI-COR, Nebraska). The PTR-ToF-MS is a chemical ionization mass spectrometer that has been developed for the continuous measurement of volatile organic compounds with limits of detection below parts per trillion (pptv) [34]. PTR-ToF-MS has been used in several applications such as the atmospheric sciences [35], food profiling [36, 37], human biomarkers [38], and the detection of explosives and chemical warfare agents (CWAs) [39]. General descriptions of the mechanism of detection of the PTR-ToF-MS are provided in prior studies [34, 40]. Briefly, energized hydronium ions are used to charge analytes in the ambient air through a non-dissociative proton transfer in the drift chamber of the instrument. The PTR-ToF-MS can detect a wide range of organic and inorganic gases (e.g., carboxylic acids, carbonyls, and aromatic hydrocarbons) that have a proton affinity greater than that of water (691 kJ/mol). The transfer of hydronium ions to VOCs occurs as follows:

1 $$\:{\mathrm{H}}_{3}{\mathrm{O}}^{+}+\mathrm{V}\mathrm{O}\mathrm{C}\to\:\:{\mathrm{H}}_{2}\mathrm{O}+\mathrm{V}\mathrm{O}\mathrm{C}{-\mathrm{H}}^{+}\:$$

The PTR-ToF-MS was operated at a drift tube pressure of 2.6 mbar and a temperature of 80 °C, with an E/N value of ~ 119 Townsend. The drift voltage (U_drift_) and inlet flow rate were set at 480 V and 200 sccm. The mass range extended to 500 m/z with a single spectrum time of 1000 ms, an extraction time of 2.0 µs, and a maximum flight time of 37.3 µs. IoniTOF software (version 4.0.97) was used for data acquisition Each h5 raw file represents a 1-hour duration. The PTR-ToF-MS used here is equipped with an ION-BOOSTER funnel and hexapole ION-GUIDE, which enhanced the sensitivity to 2000 cps/ppb and achieved a mass resolution of 6500 m/Δm. The TOF mass scale is calibrated using the Permeation Source for Calibration (PerMaSCal) unit, which is a built-in, temperature-control chamber with diiodobenzene (C_6_H_4_I_2_). The calibration peaks used were C_6_H_4_I_2_-H+ (m/z 330.848), C_6_H_4_I-H+ (m/z 203.943), and C_3_H_6_O-H+ (m/z 59.049).

The parts per trillion detection limit and high mass resolution capabilities of the PTR-ToF-MS yielded hundreds of compounds, thus requiring proper and efficient data curation and handling. The post-processing of the raw files from the PTR-ToF-MS was performed using an IONICON Data Analyzer (IDA). The software provides baseline and timing correction, peak shape determination, and mass scale–TOF calibration. More importantly, the IDA provides users with automatic and rapid extraction of hundreds of minor compounds through high-resolution peak analysis with subsequent chemical formula identification and data quantification. The application of IDA is vital in investigating hundreds of minor compounds emitted by the plant, which is a tedious process when using the manual routine. Supplemental chemical information of the ions were determined using ChemCalc, which is an online GUI that provides web services related to mass spectrometry, namely isotopic distribution simulation, peptide fragmentation simulation, and molecular formula determination. With the input of monoisotopic mass from the user, ChemCalc propose molecular formula with corresponding theoretical mass, error difference between experimental and theoretical in terms of ppb and mDa, and degree of unsaturation [41].

The LI-6800 is an infrared gas analyzer PPS that is commonly used and commercially available. It is an open gas exchange system that measures the exchange of CO_2_ and release of H_2_O in a controlled environment leaf cuvette. Gas exchange measurements were conducted using a LI-COR Multiphase Flash Fluorometer (6800–01 A) using the “Gas Exchange Only” settings to avoid interactions between the high-intensity flashes from the fluorometer and BVOC emissions. We installed either the 6 cm^2^ or 2 cm^2^ leaf area aperture, depending on experimental needs from the size of the leaf.

To remove the influence of BVOCs present in the ambient air, an external zero air (N_2_ + O_2_) supply was connected to the LI-6800 through the Filter Cover Air Inlet at the back of the console. A ¼” PTFE tubing was installed to the air inlet through an M5 hose barb. The flow from the zero-air tank was controlled with a two-stage regulator and mass flow controller set at 3.0 L/min. A Supelpure™ HC Hydrocarbon Trap was added in the line to further reduce the concentration of hydrocarbons introduced to the LI-6800. Lastly, the zero air was directed to an exhaust/T-fitting vent to prevent overpressure from excess flow entering the Li-6800.

**Fig. 1:**
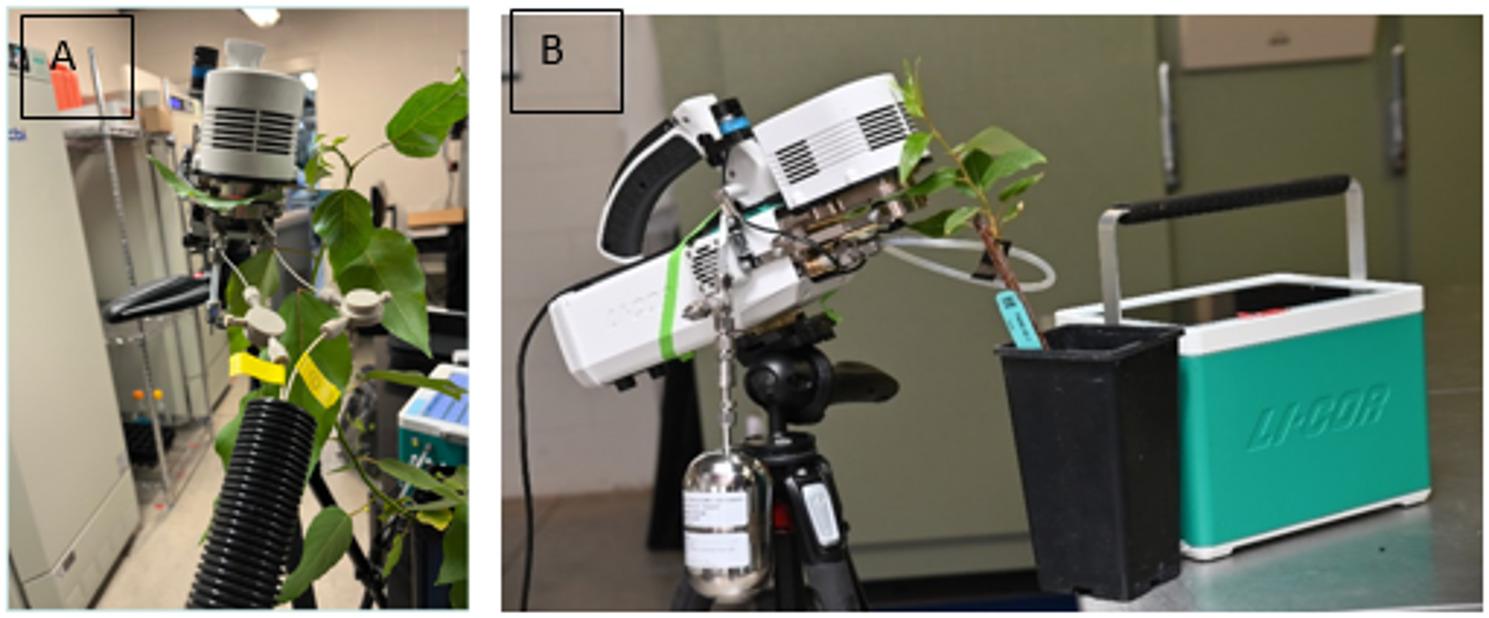
Example coupling of the LI-6800 with the PTR-ToF-MS (**A**). Setup for the collection of leaf-level VOC emission using a stainless steel canister (**B**)

The two instruments were connected using a series of fittings (brass hose and Swagelok) and tubing (PEEK and PTFE; Fig. 1). The PTFE tubings were covered with aluminum foil to prevent possible UV-induced oxidation, particularly during measurements with abundant sunlight. Most experiments performed here connected both the sample and reference exhaust port of the LI-6800 with the PTR-ToF-MS. However, the reference line did not accurately represent a blank because the reference flow bypassed the chamber, which can also emit BVOCs even without a leaf present. Thus, the data presented here were measured primarily from the sampling port.

**Fig. 2:**
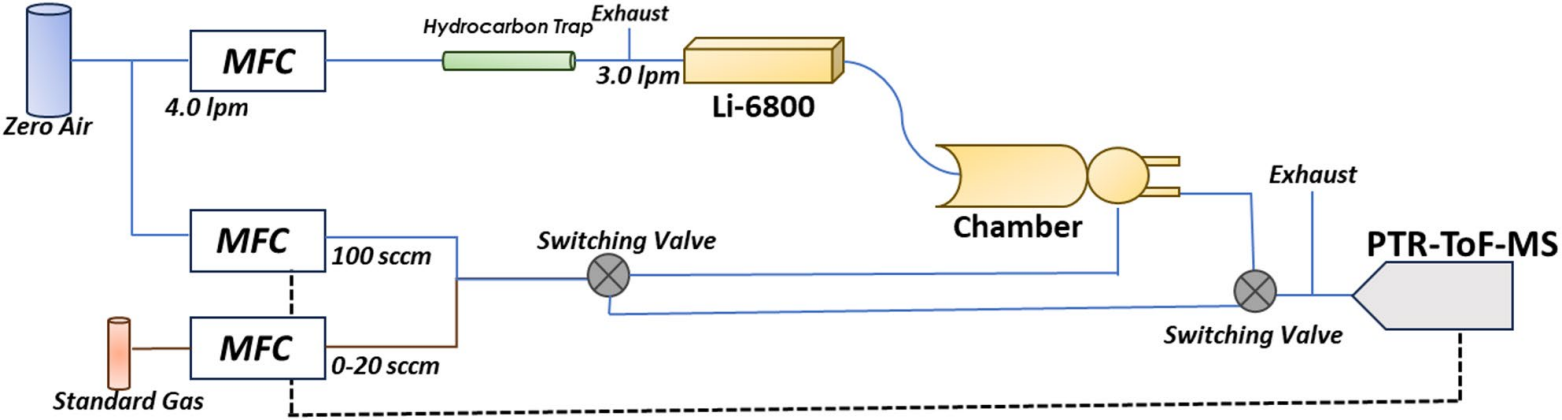
Schematic diagram of the analysis of the emission from the blank and response of the instrument to gas standards. Legends: MFC – Mass flow controller; Zero-air and Standard gas are canisters with volumes of 9000 L (Airgas, Inc.) and 98 L (Restek Corporation) at standard temperature and pressure, respectively. The dotted line between PTR-ToF-MS and the standard gas/flow manifold denotes the sequence control of the MFCs through the mass spectrometer

### Investigation of efficiency of tandem instrumentations: blank and standard analyses

According to the material datasheet (Airgas, Inc.), the zero-air gas has 100 ppb of hydrocarbon impurities that might include methane and other VOCs. Moreover, the fluorometer chamber consists of gaskets and O-rings that might contribute to the VOCs measured by the PTR-ToF-MS. To quantify these background LI-6800 and zero-air gas BVOC emissions, we performed blank analyses by altering conditions within the LI-6800 in the absence of plant material. Background analysis was performed by introducing ultra-high-purity zero air to the LI-6800, and blank temperature response curves were constructed at 20, 25, 30, 35, 40 °C. Vapor pressure deficit (VPD) was controlled at 2.5–4.5 kPA; lower values could not be achieved under high chamber temperatures. Thus, flow was set to high, at 700 µmol air s^− 1^. Three temperature response curves were collected in the dark and light; light was controlled at 1200 µmol m^− 2^ s^− 1^, which was determined to be saturating based on light response curve (data not shown). We measured one humidity response curve by controlling chamber humidity at 0, 15, 30, 45, and 60%. Humidity response measurements were conducted under controlled conditions: light at 1200 µmol m^− 2^ s^− 1^, temperature at 25 °C, and flow at 700 µmol m^− 2^ s^− 1^. Blank temperature, humidity, and light analyses were conducted with the LI-6800 foam gaskets. We further tested the background VOCs using both the LI-6800 foam and Advanced Polymer gasket sets.

The IDA software provides VOC concentrations ppb. We calculated VOC emission rates as:

2 $$\:{C}_{P}=\:{C}_{C}\:\times\:\:\frac{{Q}_{C}}{{Q}_{P}}$$

where C_P_ mixing ratio of the VOC from the sample outlet of the PPS (mol mol^− 1^), C_C_ is the identified VOC (mol mol^− 1^), Q_C_ is the flow pulled into the PTR-ToF-MS, and Q_P_ is the flow out of the PPS sample outlet. The VOC emission rate is calculated as:

3 $$\:{E}_{VOC}\:=\:\frac{{C}_{P}\:\times\:\:{Q}_{L}}{S}$$

where E_VOC_ is the VOC emission rate (nmol m^− 2^ s^− 1^), QL is the leaf chamber flow (µmol s^− 1^), and S is the leaf area inside the PPS chamber (m^2^).

The tandem of instrumentation was further characterized by introducing standard compounds with known concentrations (Fig. 2). The mixture of the gases consisted of acetonitrile, acetaldehyde, isoprene, benzene, toluene, styrene, chlorobenzene, trimethylbenzene, α-pinene (monoterpene), dichlorobenzene, and trichlorobenzene (Restek Corporation). The list of compounds comprised hydrocarbons (e.g., isoprene and toluene), oxygenated hydrocarbons (e.g., acetaldehyde), nitrogenated compounds (e.g., acetonitrile), and chlorinated hydrocarbons (e.g., chlorobenzene), which highlights the diverse molecular backbone of the representative gas standards. The diluted standard gas was introduced to the LI-6800 through 1/16” PEEK tubing placed inside the chamber. Gas plumbed directly into the LI-6800 and PTR-ToF-MS was controlled using stainless steel series three-way ball switching valves with ¼ in. Swagelok tube fitting (Swagelok Company, Solon, OH, USA). Both the standard concentration flow and zero-air gas flow (low: 0.33 L/min and high: 0.832 L /min) entering the chamber were modified to have a wide concentration range, which was utilized to evaluate the efficiency of the tandem of instrumentation.

### Application 1: emissions across pennycress genotypes

To demonstrate the tandem coupling’s use in a biologically relevant system, we surveyed the detectable mass spectra from *Thlaspi arvense*, pennycress, an emerging oilseed crop with potential as a biofuel source [42]. We assessed the variation of VOC emissions across leaves of 20 different pennycress genotypes collected from different geographic regions across the world to test that the instrumentation can detect emission variations due to differences in genotypic make-up even without the presence of plant stressors. Pennycress was grown from seed for 2.5 weeks in a greenhouse with a 25/22°C 16 h day/night cycle, with 4–8 replicates per genotype. Tandem photosynthesis–VOC measurements were conducted on individual pennycress leaves using the 2 cm LI-6800 chamber attachment, where a smaller aperture was used to fully cover the leaf area within the chamber. Leaf temperature was kept stable at 23 °C, the chamber relative humidity was controlled at 60%, reference CO_2_ was maintained at 415 ppm, light was set to saturating at 1200 µmol m^− 2^ s^− 1^ (as determined through light response curves), and flow was maintained at 600 µmol s^− 1^. Coupled photosynthesis–VOC measurements were collected after the leaf stabilized to chamber conditions. Once conditions were stable, VOC data were collected continuously for three minutes. The photosynthetic rate was logged at the end of the VOC measurements, and individual VOC point measurements were taken as the average of the 3 min time period.

### Application 2: comparison of BVOC emission from poplars with varying soil media

Besides the direct connection between the two instruments, the LI-6800 was also attached to a stainless-steel canister for the offline collection of VOCs emitted by leaves (Fig. 1B). This setup is beneficial for the field collection of VOCs performed at sites/locations where an PTR-ToF-MS instrument cannot be deployed due to lack of appropriate facilities. The setup was demonstrated by surveying the BVOCs from the leaf-level emission of *Populus trichocarpa* with different genotypes and soil media (i.e., potting mix versus field soil). The use of different soil media with varying compositional differences and nutrient availability is expected to impact the plants’ physiological functions, which influence the emission of BVOCs. Similar to pennycress, the genetic makeup of different poplar species might dictate their inherent capacity to produce and emit BVOCs. The poplars analyzed here consisted of five different genotypes with three replicates for both soils, totaling 30 plants cultivated and studied.

VOCs were collected using 400 mL electropolished stainless steel miniature air sampling canisters with quick-connect stem fitting (Restek Corporation, Bellefonte, PA, USA). The canisters are also equipped with female RAVEqc valve to 1/4” male compression fitting. The intake vacuum flow of the canister was controlled using a mass flow controller at a flow of 80 sccm, which allowed 5 min of integrated collection procedure. Prior to sampling, the canisters were conditioned by three cycles of evacuation ( < − 29 inHg), zero-air purging, and heating in an oven at 120 °C. If no electrical power is available (i.e., field measurement), a sampling kit with a critical orifice can be used to limit the intake flow of the canister. The VOC measurement of the contents of the canisters was implemented by connecting the canister to the PTR-ToF-MS using a series of PTFE tubing and solenoid valves.

Tandem photosynthesis–VOC measurements were conducted on individual poplar leaves using the 6 cm LI-6800 chamber attachment. Leaf temperature was kept stable at 25 °C, the chamber relative humidity was controlled at 60%, reference CO_2_ was maintained at 415 ppm, light was set to saturating at 1500 µmol m^− 2^ s^− 1^ (as determined through light response curves), and flow was maintained at 500 µmol s^− 1^. Coupled photosynthesis–VOC measurements were collected after the leaf stabilized to chamber conditions.

### Development of VAPOR: an automated procedure for VOC analysis

The IDA software is highly efficient in identifying and resolving hundreds or even thousands of ions. These compounds originate from the target samples, such as vegetation and leaf emissions, or from background interference caused by the dilution air, tubing materials (such as PEEK and PFA), and instrument internal components. The identification and quantification of many relevant compounds are time-consuming for users, which limits the time available for in-depth scientific analysis of the relationship between plant physiology and biogenic VOC emissions. To significantly reduce the time required for manual calculations, the VOC Analysis and Processing Optimization Resource (VAPOR), a publicly accessible algorithm, was developed. This tool aids researchers in managing large volumes of high-resolution mass and temporal data generated by PTR-ToF-MS analysis.

VAPOR is designed to provide users with a list of relevant VOCs based on their criteria and the average concentrations of those compounds. The algorithm also alerts users to any instrument failures that may affect the data collected from specific plant samples. VAPOR is a key component of Oak Ridge National Laboratory’s (ORNL’s) INTERSECT initiative, which aims to facilitate automated workflows across various scientific disciplines. This initiative focuses on the development of data management software, tools to enable data analysis workflows, and experiment management software.

The current version of the VAPOR algorithm targets the analysis of large numbers of plant samples, particularly for applications such as genome-wide association studies. Future iterations will include emission analyses related to microbiome studies, such as the interactions of bacterial and fungal systems. VAPOR will be integrated into Automated Control Testbed for Integration, Verification, and Emulation (ACTIVE) framework, which will allow users to visualize results over a web-based graphical user interface. ACTIVE is an operations management framework within ORNL with a forthcoming open-source release designed to facilitate the testing and deployment of software for control of automated facilities.

The current version of VAPOR performs three specific tasks:

*1.* *Downscaling of Ions*: The hundreds or thousands of ions generated by IDA are selected based on three criteria:

- The concentration of the analyte must exceed the limit of detection, which is defined as the sum of the average blank level plus three times the standard deviation of the blank.
- The concentration must surpass 5.0 ppt, the conservative limit of detection for PTR-ToF-MS.
- The concentration of compound across all plant samples must exceed a threshold standard deviation value (0.1 by default).

Ions are selected and reported on both a per-plant (meeting both criteria A and B) and across-plant (criterion C) basis.

2.  *Immediate Reporting of Average Concentration of All Compounds*: The IDA exports a single file containing the time series data for all compounds. The calculation process corrects each compound’s concentration by removing contributions from the blanks’ measurements. The algorithm outputs concentrations in a CSV file for ease of use.
3.  Instrument Performance Evaluation: Genetic and plant physiological studies typically require hundreds of samples to accurately identify genetic variants associated with specific diseases or traits. Due to time constraints and the large volume of samples, instrument issues such as blockages at the inlet or unexplained decreases in reagent ion signals can go undetected during the analysis of the plants. To inform users of any irregularities in instrument performance, VAPOR evaluates parameters such as pressure controller (PC) values and the raw counts of the reagent ion at mass 21.0221. The algorithm reports on specific plant samples whose performance deviates beyond a threshold value (10% of the typical value), thereby indicating which samples need to be reanalyzed to ensure high-quality data for scientific research.

Further information and instructions can be found in the documentation and reference implementation associated with the algorithm. The open-source code for VAPOR is accessible at https://github.com/INTERSECT-BESS/ORNL-VOC. In this study, VAPOR was used to post-process the VOC results generated from the offline collection of gases from poplars with different soil media.

### Statistical analyses

Blank analysis of isoprene response to temperature and light was performed using an analysis of covariance (ANCOVA) model, where light conditions were treated categorically as either “light” or “dark.” Genotypic differences in pennycress methanol emissions were compared using a mixed-effects model, where genotype is the fixed effect and replicate is the random effect. Replicates were treated as a random factor because individual replicates were all measured on the same day. Methanol response to *A*_*23*_ and *g*_*s*_, individual, was also analyzed using a mixed model. A₂₃ is the net CO₂ assimilation rate measured at 23 °C. gₛ is the stomatal conductance to water vapor, which represents water vapor (and indirectly CO₂) movement through the stomata. Mixed-effects models were analyzed using the “lmer” function of the “lme4” package [43] in R version 4.5.2 (R Core Team). *Post hoc* separation of means between genotypes was performed using a Type II Wald chi-square test with the “emmeans” function in the “emmeans” package (Lenth, 2023). Comparisons of isoprene concentration across poplar lines grown in different soil media were compared using an ANOVA model. Tukey tests were used for *post hoc* separation of means.

## Results

### Background measurements and standard analysis

Using the IDA, more than 1,300 ions were identified during the blank analysis conducted under light conditions. Among these, we focused on the top 20 dominant ions (Table 1). All of these ions shared a common hydrocarbon backbone structure (C_x_H_y_), notably including the C_5_H_9_^+^ ion, which corresponds to the protonated isoprene compound. To investigate the effects of varying environmental conditions, the blank analysis was repeated under dark conditions, as well as at elevated relative humidity and temperature (Table 2). While the major ions detected were similar across conditions, certain ions were common to both dark and light conditions. In agreement with the results under light conditions, isoprene remained one of the dominant ions in the dark. Most of the other compounds also retained a hydrocarbon backbone. Overall, both analyses indicated minimal interference from background VOCs.

**Table 1:** List of dominant ions measured in the blank analysis with light turned on inside the 25 °C chamber

Rank	Exp. Mass [M + H]	MF	Theor Mass	mDa	DU	Rate. (mmol m^− 2^ s^− 1^)	Notes
1	41.038	C_3_H_5_^+^	41.039	−1.03	1.5	0.732 ± 0.399	
2	55.053	C_4_H_7_^+^	55.055	−2.15	1.5	0.477 ± 0.086	
3	39.023	C_3_H_3_^+^	39.024	−0.71	2.5	0.456 ± 0.271	
4	69.068	C_5_H_9_^+^	69.070	−2.37	1.5	0.194 ± 0.032	Isoprene
5	95.081	C_7_H_11_^+^	95.086	−4.85	2.5	0.175 ± 0.024	
6	81.068	C_6_H_9_^+^	81.070	−2.28	2.5	0.159 ± 0.024	
7	57.069	C_4_H_9_^+^	57.070	−1.91	0.5	0.190 ± 0.104	
8	109.097	C_8_H_13_^+^	109.102	−4.73	2.5	0.098 ± 0.013	
9	83.083	C_6_H_11_^+^	83.086	−3.3	1.5	0.97 ± 0.016	
10	43.053	C_3_H_7_^+^	43.055	−1.45	0.5	0.203 ± 0.166	
11	67.052	C_5_H_7_^+^	67.055	−2.42	2.5	0.085 ± 0.014	
12	18.035	H_2_O^+^	18.011	24.36	0	0.497 ± 0.462	

MF – Molecular formula; mDa– Absolute difference of experimental (Exp) m/z and monoisotopic (Theor) mass in terms of milliDalton and parts per million; DU – Degree of unsaturation; Conc – Average concentration and standard deviation of the ions among the three replicates. Rate is the calculated emission rate of each VOC based on a previous study [28]

**Table 2:** List of major ions measured in the blank analysis with dark conditions. Legend description can be found in Table 1

Rank	Exp. Mass [M + H]	MF	Theor Mass	mDa	DU	Rate. (nmol m^− 2^ s^− 1^)	Notes
1	37.029	H_3_OH_2_O^+^	37.028	−1		0.278 ± 0.150	
2	55.053	C_4_H_7_^+^	55.055	0.02	1.5	0.408 ± 0.287	
3	69.068	C_5_H_9_^+^	69.070	−2.37	1.5	0.104 ± 0.045	Isoprene
4	81.068	C_6_H_9_^+^	81.070	−2.28	2.5	0.114 ± 0.063	
5	109.097	C_8_H_13_^+^	109.102	−4.73	2.5	0.065 ± 0.030	
6	67.052	C_5_H_7_^+^	67.054	−2.42	2.5	0.052 ± 0.026	
7	97.098	C_7_H_13_^+^	97.101	−4.13	1.5	0.042 ± 0.025	

**Fig. 3:**
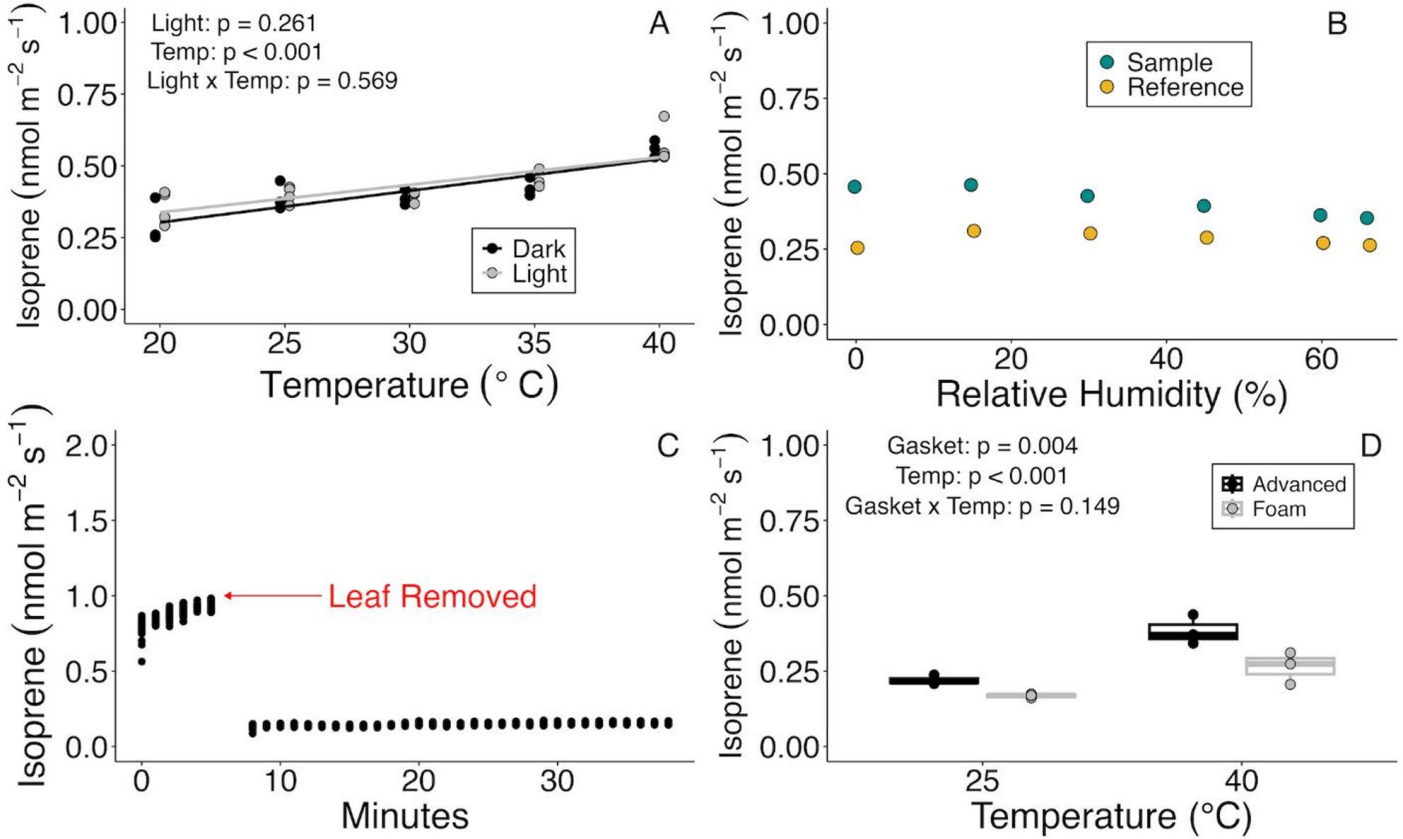
Concentration of isoprene across different temperatures in light and dark conditions (**A**), response to relative humidity (**B**), demonstration of the immediate drop of isoprene concentration following leaf removal from the chamber (**C**), and comparison between LI-6800 gasket types (**D**). Note The difference in the isoprene blank presented in Tables 1 and 2 with panel A is accounted to the instrument performance when these experiments were performed

The blank analysis revealed that the background concentration of isoprene increased with rising temperatures. Additionally, it showed no significant difference in isoprene emissions between light and dark conditions (Fig. 3A). At an elevated temperature of 40 °C, the concentration of isoprene (0.57 nmol m^− 2^ s^− 1^) was higher compared to that at 20 °C (0.33 nmol m^− 2^ s^− 1^). This trend was consistent under both light and dark conditions. The relative humidity in the chamber was adjusted from dry (0%) to wet (65%) to evaluate the impact of moisture on the detection of background isoprene (Fig. 3B). Measurements from both the sample and reference chambers indicated that background isoprene levels were not affected by chamber humidity. Furthermore, when the leaf inside the chamber was removed, only minimal isoprene emissions were detected (Fig. 3C), suggesting negligible memory effects of isoprene between biological samples. Blank analyses conducted with different LI-6800 gasket sets revealed that the foam gasket exhibited significantly lower background isoprene emission rates compared to the advanced polymer gasket. Specifically, the foam gasket produced 27% and 37% less background isoprene at 25 °C and 40 °C, respectively (Fig. 3D).

**Fig. 4:**
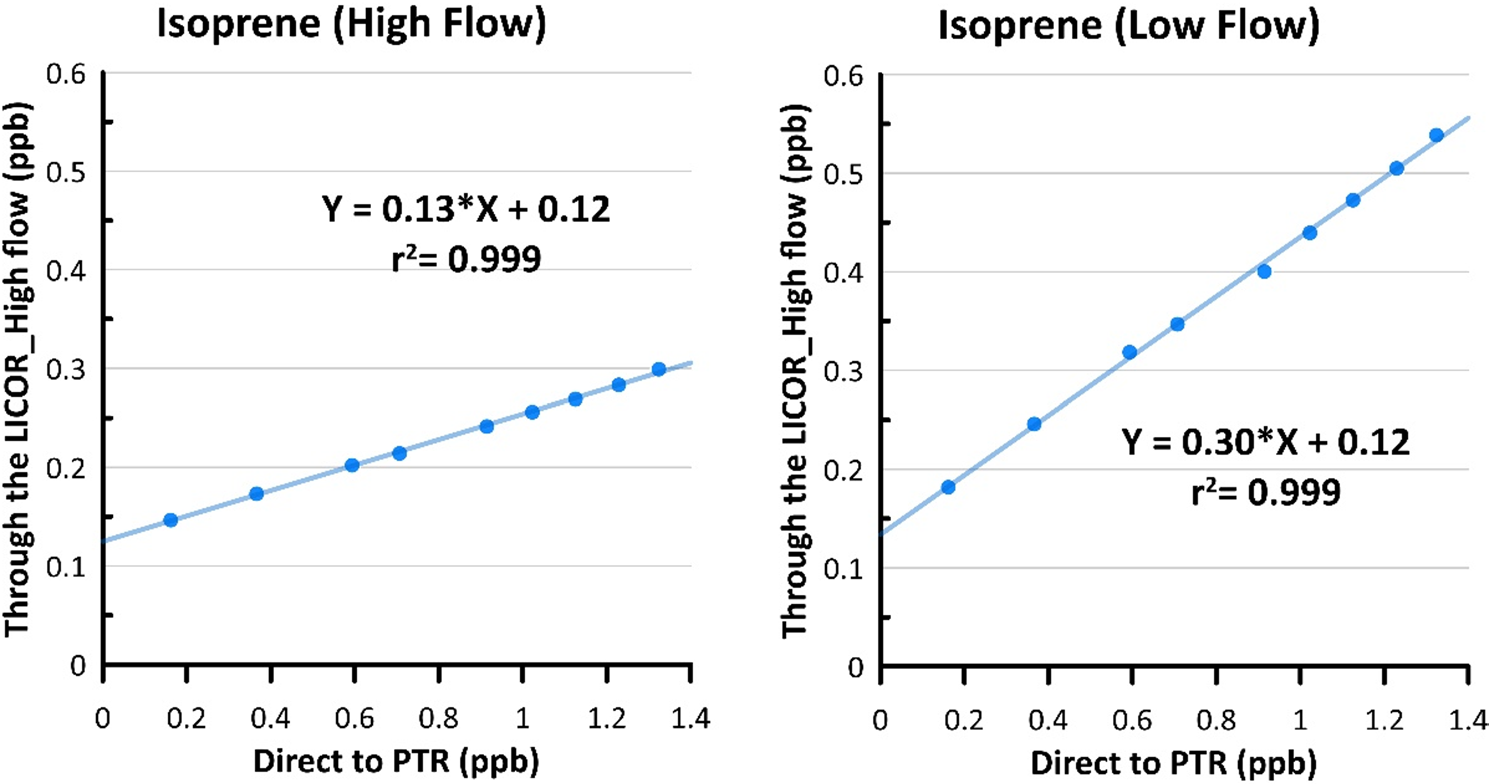
Correlation plot of the standard concentration of isoprene (direct to PTR) and measured isoprene from LI-6800 standard port exhaust (through the LICOR). The left panel shows results under high chamber flow, and the right panel shows results under low chamber flow

To evaluate potential leaks and other loss mechanisms (e.g., wall deposition and memory effects) in the tandem LI-6800–PTR-ToF-MS system, we introduced a mixture of gases with varying known concentrations. There was a strong correlation between isoprene concentrations measured at the LI-6800 sampling port exhaust and those measured directly from the standard gas tank into the PTR-ToF-MS system (r² > 0.99, Fig. 4). The calculated ratio of the slopes (2.34) of the best-fit lines for low and high flow rates was consistent with the chamber flow ratio (2.52). Furthermore, the identical *y*-intercept values of the linear regression curves indicate a constant background concentration of isoprene regardless of the flow rate introduced into the chamber. This pattern was similarly observed for ten other standard gases with varying numbers of carbon, hydrogen, and chloride atoms (Table 3). These findings validate the system’s integrity and its resistance to leakage or absorption. Based on the results of both the blank and standard analyses, the LI-6800–PTR-ToF-MS tandem system demonstrates an effective capability for measuring leaf-level emissions and physiological parameters. The system exhibits a highly linear response to VOC concentrations, minimal impurity interference, and negligible VOC losses from either leakage or chamber wall interaction.

**Table 3:** Regression statistics for the relationship between standard concentrations of other VOCs and measurements from the LI-6800 under high- and low-flow chamber conditions

Standard Gas	High flow inside chamber			Low flow inside chamber		
	Slope	*r* ^2^	y-intercept	Slope	*r* ^2^	y-intercept
Acetonitrile (C_2_H_3_N)	0.120	0.998	0.092	0.283	0.985	0.079
Acetaldehyde (C_2_H_4_O)	0.097	0.996	3.814	0.302	0.996	3.837
Benzene (C_6_H_6_)	0.106	0.999	0.066	0.242	0.999	0.051
Toluene (C_7_H_8_)	0.105	0.997	0.323	0.224	0.998	0.490
Styrene (C_8_H_8_)	0.100	0.995	0.029	0.189	0.988	−0.001
Benzaldehyde (C_7_H_6_O)	0.097	0.997	0.054	0.132	0.996	0.050
Chlorobenzene (C_5_H_5_Cl)	0.108	0.995	0.032	0.223	0.994	0.033
Trimethylbenzene (C_9_H_12_)	0.090	0.995	0.027	0.147	0.988	0.018
Monoterpene (C_10_H_16_)	0.107	0.997	0.052	0.221	0.994	0.032
Dichlorobenzene (C_6_H_4_Cl_2_)	0.067	0.997	−0.006	0.088	0.986	−0.008
Trichlorobenzene (C_6_H_3_Cl_3_)	0.026	0.957	0.002	0.024	0.897	0.001

### Utilizing tandem instrumentation for large-scale plant VOC measurements

**Fig. 5:**
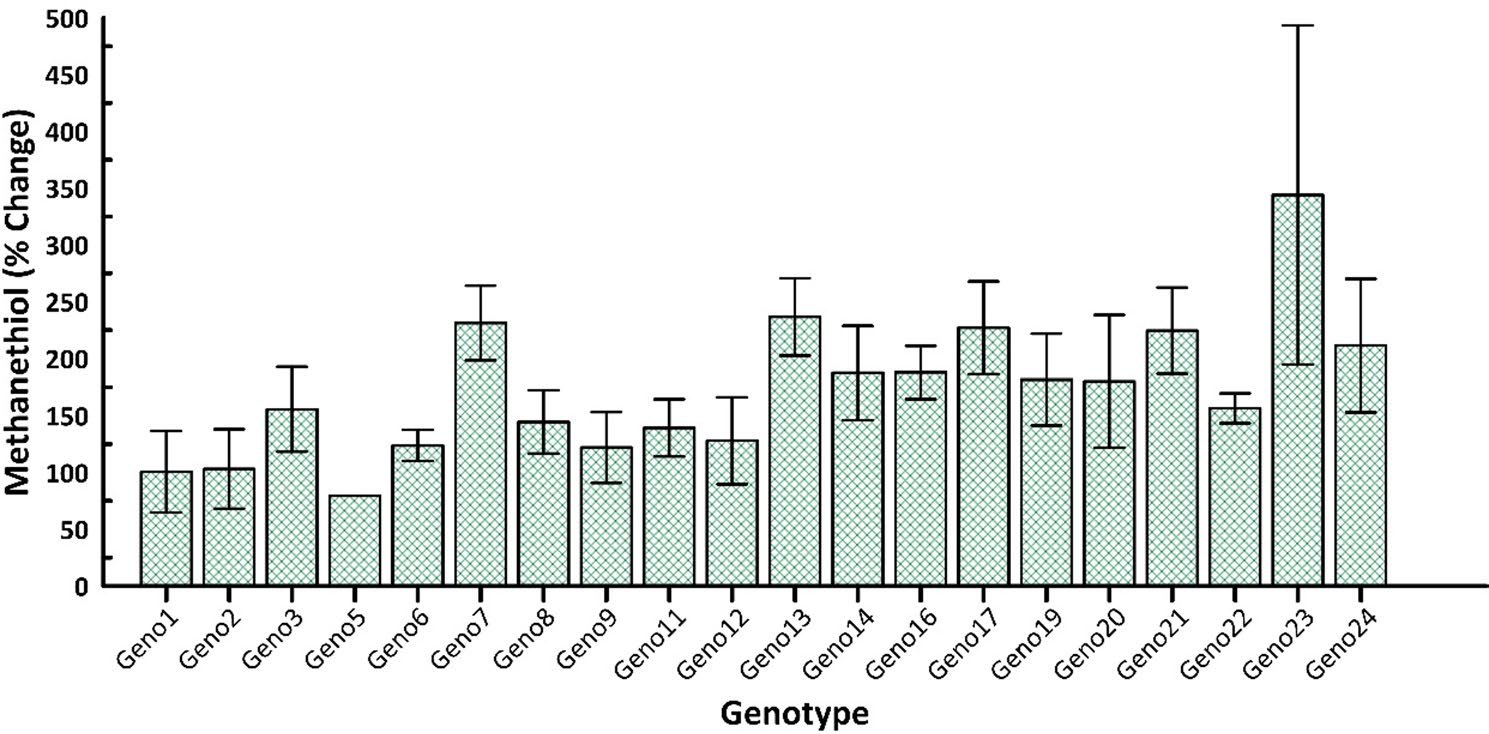
Average percent increase of methanethiol compared to blank observed from pennycress with different genotype. The error bars are represented by standard error (*n* = 4–8)

A comprehensive survey of the mass spectra was performed using the IDA on pennycress leaf emissions. The survey revealed several intriguing ions corresponding to VOCs that play crucial roles in and contribute significantly to plant physiology. Among these, methanethiol (CH_3_SH) was consistently detected across all genotypes, albeit at varying concentrations. Methanethiol was observed at a mass-to-charge ratio of 49.012 (Fig. 5).

**Fig. 6:**
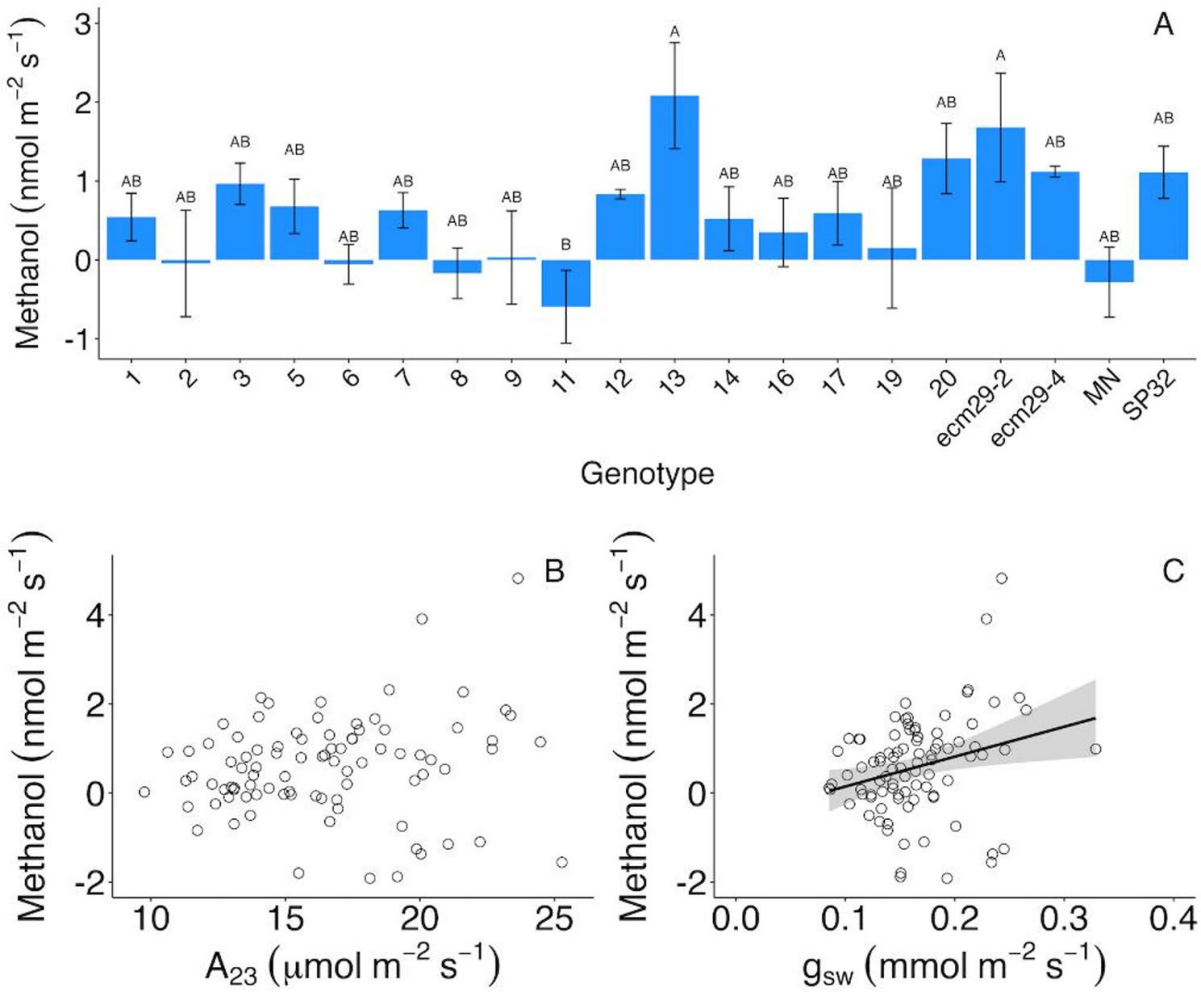
Methanol emissions (nmol m^− 2^ s^− 1^) at 23 °C and compared across genotypes (A). Correlation between methanol emissions (nmol m^− 2^ s^− 1^) and photosynthesis (µmol m^− 2^ s^− 1^) at 23 °C (A_23_, B) and stomatal conductance (g_sw_, mmol m^− 2^ s^− 1^,C ). Error bars represent standard error (*n* = 4–8). Note that the negative rates are considered blank values, which are within the error of instrument. Letters (A, B, and AB) above the bar graph depict results of posthoc separation of means, where different letters represent significantly different Methanol emission rates (Tukey, *p* < 0.05)

Another notable VOC detected during the pennycress leaf-level analysis was methanol (CH_3_OH). By directly integrating the PTR-ToF-MS with the photosynthesis system, we were able to examine the relationship between methanol emissions and photosynthetic activity (A_23_) and stomatal conductance (Fig. 6). The analysis revealed no significant correlation between methanol and photosynthesis.

(*p* = 0.205, R^2^_marginal_ = 0.022, R^2^_conditional_ = 0.070). However, methanol emissions and stomatal conductance showed a statistically significant positive correlation (*p* = 0.012, R^2^_marginal_ = 0.077, R^2^_conditional_ = 0.137), though the observed trend was weak and may lack biological significance.

### Offline VOC collection via canisters: soil type effects on VOC emissions

We utilized stainless steel canisters to collect VOC samples for a comparative analysis of BVOC emissions across various *Populus* genotypes and soil types. Similar to the findings in pennycress, the analysis of 30 poplar plants sampled using offline canisters revealed over 100 ions with signal intensities exceeding those observed in the blank canister. Using the VAPOR algorithm, the average concentrations of all BVOCs were rapidly determined, enabling straightforward identification of the major compounds emitted at the leaf level. VAPOR also indicated that there were no instrumental irregularities, based on PC and reagent ion counts, during the measurement of the VOCs from the canisters. All the information and routine output for all the BVOCs generated by the VAPOR are provided in the supplement file. Additionally, VAPOR determined the frequency of samples in which BVOC concentrations surpassed the predefined criteria set in the algorithm (“Frequency”). VOCs detected in poplar emissions included a mix of pure hydrocarbons (e.g., isoprene), oxygenated compounds (e.g., acetaldehyde), and nitrogen-containing compounds (e.g., allylamine and ethanimine) (Table 4). Among these VOCs, acetaldehyde exhibited elevated concentrations at the leaf level; however, only 20% of the plant samples met the VAPOR algorithm’s criteria. In contrast, compounds with a proposed protonated molecular formula of C_3_H_8_N^+^ were detected at relatively low concentrations but had high occurrence rates (70%) across all poplar samples. The VAPOR algorithm delivers critical insights into VOC emissions, assisting users in identifying which compounds warrant further investigation among the hundreds detected by the highly sensitive instrument.

**Table 4:** Some of the compounds detected from leaf emission of poplar

Exp. Mass [M + H]	MF	Theor Mass	Emission rate (nmol m^− 2^ s^− 1^)	Frequency	Possible Compounds
44.050	C_2_H_6_N	44.050	0.067215	67%	Ethanimine, N-Methylmethanimine
**45.034**	**C**_**2**_ **H**_**5**_ **O**	**45.034**	10.08871	**20%**	**Acetaldehyde**
58.066	C_3_H_8_N	58.065	0.08642	70%	Allylamine
63.044	C_2_H_7_O_2_	63.044	0.21765	20%	Ethylene glycol, Methoxymethanol
**69.070**	**C**_**5**_ **H**_**9**_	**69.070**	1.843621	**100%**	**Isoprene**
89.062	C_4_H_9_O_2_	89.060	0.428898	33%	Butyric acid, Ethyl acetate
103.078	C_5_H_11_O_2_	103.075	0.240055	60%	Valeric acid, Isopropyl acetate
107.068	C_4_H_11_O_3_	107.071	0.118427	27%	Butanetriol
117.057	C_5_H_9_O_3_	117.055	0.038409	67%	Levulinic acid

Frequency **–** The percentage of plants emitting a given BVOC that satisfied the conditions set in VAPOR. For instance, isoprene has 100% frequency, which means that all plants, regardless of soil media, emitted detectable isoprene

**Fig. 7:**
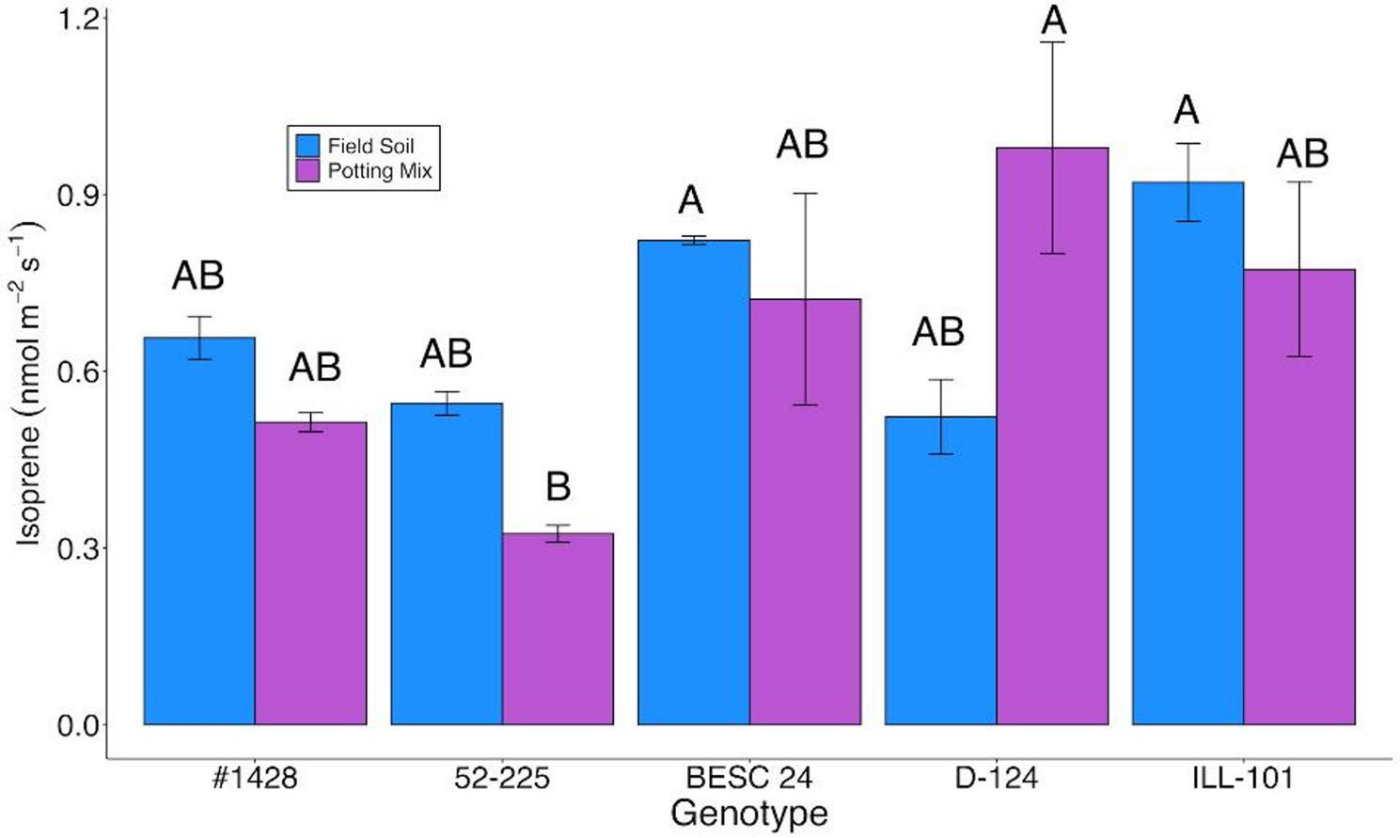
Isoprene emission rate observed from different poplar genotypes grown in different soil media. Error bars are represented by standard error (*n* = 3). Letters (A, B, and AB) above the bar graph depict results of posthoc separation of means, where different letters represent significantly different isoprene emission rates (Tukey, *p* < 0.05)

In our study, the most notable BVOC detected from the leaf-level emissions was isoprene, which displayed an average concentration of 0.68 nmol m^− 2^ s^− 1^ and consistently exceeded VAPOR’s criteria across all the plants analyzed. ANOVA analysis revealed a significant interaction between soil type and genotype (*p* = 0.016), and a *post hoc* Tukey test identified several key differences across treatments. Specifically, the 52–225 genotype grown in potting mix emitted lower levels of isoprene compared to IL-101 and BESC-24 genotypes grown in field soil, as well as D-124 grown in potting mix (Fig. 7). These results suggest that both soil type and genotype influence isoprene emissions, underscoring the complex interplay of environmental and genetic factors in BVOC production by poplars.

## Discussion

### Low background emissions, reduced carryover, and minimized leakage risks

Our analysis of the tandem LI-6800 and PTR-ToF-MS instrumentation under varying environmental conditions revealed minimal concentrations of background BVOCs. Notably, we detected a consistent background concentration of isoprene, a major BVOC emitted by many plants in response to biotic and abiotic stress. The presence of background isoprene likely results from its adherence to chamber components, such as polyethylene and neoprene gaskets, or other surfaces inside the chamber. This conclusion is further supported by evidence showing that the type of gasket used influences the measured background isoprene levels. For users seeking to minimize background values, we recommend using the LI-6800 foam gaskets rather than the advanced polymer gaskets.

Despite the detection of background isoprene, its concentrations under light conditions and similar temperature conditions were, on average, lower than the concentrations observed during our poplar experiments with leaves in the chamber (below 0.45 nmol m^− 2^ s^− 1^), indicating minimal interference during actual sample analysis. However, background isoprene concentrations increased under increased temperature conditions, both in light and dark measurements. This rise in isoprene concentration with temperature is likely a result of the heating of chamber components, such as gaskets and walls, leading to the release of more VOCs. Evident in Fig. 7 that poplar isoprene concentration ranged from 0.30 to 1.33 nmol m^− 2^ s^− 1^, which trended higher compared to the blank levels (0.25–0.67 nmol m^− 2^ s^− 1^). It is also manifested in the immediate reduction of isoprene concentration following leaf removal from the chamber shown in Fig. 3C.

Additionally, our results demonstrated that isoprene concentrations quickly returned to background levels immediately after removing a leaf from the chamber. This behavior highlights the efficiency of the tandem LI-6800–PTR-ToF-MS system in handling varying temperature conditions without carryover effects from enhanced VOC concentrations associated with previous samples. This robustness makes the system particularly well-suited for precisely measuring VOC emissions under dynamic environmental conditions.

### Enhanced instrument sensitivity and VAPOR algorithm uncover key BVOCs

Even without applying specific environmental treatments that will enhance the emission of BVOCs in our pennycress study, the coupling of the LI-6800 with the PTR-ToF-MS demonstrated remarkable sensitivity, enabling the detection of significant VOC differences across genotypes. Among the key VOCs measured, methanethiol was detected at notably high levels [9] and plays a key role in the organic sulfur cycle [44]. In the atmosphere, methanethiol reacts with hydroxyl radicals (OH) to produce the methylthiyl radical CH_3_S, which serves as a precursor for the formation of sulfur dioxide and thus contributes to acid rain [45]. The variation of the methanethiol observed across different genotypes might be critical in reducing emission of methanethiol, which subsequently impacts acid rain generation. We also found variations in methanol across our pennycress genotypes and found that leaf emissions rose with increasing stomatal opening. Methanol is emitted by most plants, particularly during the early stages of leaf expansion [46]. A similar method showed that release of biogenic methanol during daytime is co-regulated by leaf temperature and stomatal conductance [47]. However, this compound also has implications for atmospheric chemistry because methanol is the most dominant atmospheric nonmethane organic gas, which contributes to the atmospheric variation of oxidants such as ozone and OH^•^ radicals [48]. The positive correlation between stomatal conductance and methanol emission is likely due to a lower resistance of overall gas movement allowed by higher stomatal opening. However, direct methanol foliar spray can lead to increased leaf stomatal conductance and photosynthesis [49, 50] through enhanced protein regulation of guard cell opening (Zhao et al. 2014). Further studies are needed to elucidate cause and effect interactions between pennycress stomatal conductance and methanol emissions.

Our analysis of the effect of soil type on poplar VOC emissions using offline canisters revealed distinct differences across treatments. Offline VOC collection using canisters was demonstrated as a practical and effective method that can complement direct measurements from the LI-6800 coupled with the PTR-ToF-MS instrument. By leveraging the VAPOR algorithm, several VOCs were identified in the samples, and a biologically significant VOCs, isoprene, was consistently prevalent across all poplar samples. Acetaldehyde in particular is a crucial compound in plant physiology, playing a central role in stress responses. Its emissions are known to increase in reaction to various stressors, such as mechanical damage to leaves, darkness, drought conditions, and especially anoxic stress caused by oxygen deprivation [29, 31, 51–53]. In the atmosphere, acetaldehyde is one of the most dominant oxygenated VOCs and is a precursor for peroxyacetyl nitrate, which contributes to the long-range transport of NO_x_ [54–56]. The estimated annual release of acetaldehyde from plant biosynthesis into the atmosphere is significant at 213 Tg per year [57, 58].

Our measurements showed that, on average, poplar exhibited enhanced levels of isoprene above the blank measurement. We measured isoprene emissions ranging 0.33–1.33 nmol m^− 2^ s-1. These rates are comparable to Arabidopsis transformed for isoprene emissions [22], but 10–40 times lower compared to rates measured in *Populus trichocarpa* [59]. This BVOC is generated during the light-dependent metabolism in the chloroplast through the 2-C-methyl-D-erythritol 4-phosphate pathway [12]. Isoprene has a global estimate between 210 and 990 Tg C yr^− 1^, making its emissions comparable with those of methane [5]. The double bond moiety of isoprene contributes to its role in the atmospheric oxidation that generates ozone and atmospheric aerosols. This implies that the physiochemical properties of soil (e.g., nitrogen content, pH, moisture) and composition (i.e., microbial) influence the emissions of isoprene, which subsequently impact the atmospheric ozone and aerosol formation. The demonstration of coupled leaf-level measurements shown here will allow targeted leaf-level approaches to estimate critical BVOCs such as acetaldehyde and isoprene under altered environmental conditions. This capability will be highly relevant in assessing the crucial plant physiological functions under future extreme climate conditions, in which extreme temperature (> 30 °C) and elevated levels of CO_2_ will impact the distributions of atmospheric BVOCs from vegetation that will subsequently alter aerosol-cloud-climate interaction.

## Conclusion

The simultaneous characterization of the leaf-level emission of VOCs and plant physiological parameters is critical in linking vegetative genotypic differences with atmospheric chemistry, air quality, and global climate. Here, the highly sensitive PTR-ToF-MS 6000 × 2 was coupled with an LI-6800 photosynthesis system to uncover hundreds of leaf-level VOCs while monitoring photosynthesis and stomatal conductance. The VAPOR algorithm was also developed for the rapid investigation of the comprehensive data set generated by the coupled instrumentation. Blank measurements (i.e., empty chamber) under variable light and temperature revealed biologically important VOCs, such as isoprene, but at concentrations lower than those resulting from leaf emissions. Even still, the detection of these background-level VOCs suggests that frequent blank measurements should be conducted and accounted for when using this coupled instrumentation. The introduction of standard concentrations of VOCs revealed linear responses of the tandem instrumentation, which demonstrates minimal memory effects and indicates that little to no chamber flushing is needed between plant measurements. Investigation of the leaf-level emissions of poplar and pennycress plants showcased contrasting amounts of methanethiol, methane, isoprene, and other minor VOCs. The application of the tandem instrumentation and the VAPOR routine not only revealed several critical BVOCs but also indicated that targeted genetic selection of biofuel crops might be a valuable procedure in improving the level of atmospheric ozone and particles, which have implications for global warming and its feedback with vegetation. This study concludes that the tandem use of the chemical ionization mass spectrometer and photosynthesis system, complemented by the post-processing capability of VAPOR, is a valuable tool in atmospheric chemistry and plant physiology.

## Supplementary Information

Below is the link to the electronic supplementary material.

Supplementary Material 1
