# Supplementary material for: Coupling of high-resolution mass spectrometer and photosynthesis system for comprehensive leaf volatile metabolite profiling
Source: Plant Methods. 2026 May 22;22:58. doi: 10.1186/s13007-026-01531-8 (PMC13308210; doi:10.1186/s13007-026-01531-8)
Supplement: Supplementary file 1 — Supplementary Material 1 [file 13007_2026_1531_MOESM1_ESM.docx]

**Coupling of High-Resolution Mass Spectrometer and Photosynthesis System for Comprehensive Leaf Volatile Metabolite Profiling**

Kelsey R. Carter^1,#^, Christian Mark Salvador^1,#,*,^, Savana Colegate^1^, Alyssa Carrell^2^, Jun Hyung Lee^2,3^, Robert Smith^4^, Marshal McDonnell^4^, Sara Jawdy^2^, David McLennen^2^, Tyler Hackworth^2^, Lianhong Gu^1^, Melanie A. Mayes^1^, Udaya Kalluri^2^, Thomas D. Sharkey^5^, David J. Weston^2^

*^1^Environmental Sciences Division, Oak Ridge National Laboratory, Oak Ridge, TN, USA*

*^2^Biological Sciences Division, Oak Ridge National Laboratory, Oak Ridge, TN, USA*

*3Department of Environmental Biology, State University of New York College of Environmental Science and Forestry, Syracuse, NY , USA*

*^4^Computer Science and Mathematics Division, Oak Ridge National Laboratory, Oak Ridge, TN, USA*

*^5^Department of Botany, University of Wisconsin-Madison, Madison, WI 53706, USA*

*^#^Authors contribute equally*

^*^Corresponding Author: Christian Mark Salvador ([salvadorcg@ornl.gov](mailto:salvadorcg@ornl.gov))

**Sample output for VAPOR algorithm**

**Downscaling of Ions**

***Gases above threshold detected for plant 103_52-225_R1_PM***

['m/z 65.00594 []', 'm/z 65.06 []', 'm/z 69.07034 [C5H9]', 'm/z 73.06516 [C4H9O1]', 'm/z 74.9958 []', 'm/z 75.02612 []', 'm/z 76.02687 []', 'm/z 80.99988 []', 'm/z 91.06341 []', 'm/z 92.06132 []', 'm/z 93.03758 []', 'm/z 94.03747 []', 'm/z 95.01496 []', 'm/z 95.03305 [C2H7O4]', 'm/z 96.01562 []', 'm/z 105.0681 [C3H9N2O2]', 'm/z 109.0677 [C7H9O1]', 'm/z 111.0475 [C6H7O2]', 'm/z 112.0474 []', 'm/z 113.0277 []', 'm/z 114.0249 []', 'm/z 115.1108 [C7H15O1]', 'm/z 116.1102 [C6H14N1O1]', 'm/z 126.9033 []', 'm/z 129.0578 [C6H9O3]', 'm/z 129.0905 [C7H13O2]', 'm/z 131.0377 [C5H7O4]', 'm/z 133.1225 [C7H17O2]', 'm/z 143.1052 [C8H15O2]', 'm/z 149.0945 [C5H13N2O3]', 'm/z 167.0544 [C5H11O6]', 'm/z 168.0529 []', 'm/z 169.0351 []', 'm/z 169.0593 [C7H9N2O3]', 'm/z 170.0358 []', 'm/z 171.1367 [C10H19O2]', 'm/z 185.0681 [C11H9N2O1]', 'm/z 187.0437 [C11H7O3]', 'm/z 223.0551 [C18H7]', 'm/z 224.0609 []', 'm/z 225.0428 [C10H9O6]', 'm/z 226.0428 []', 'm/z 227.0336 [C13H7O4]', 'm/z 228.0331 []', 'm/z 239.0928 [C12H15O5]', 'm/z 240.0937 [C11H14N1O5]', 'm/z 241.0752 [C11H13O6]', 'm/z 242.0748 [C14H12N1O3]', 'm/z 243.0539 [C10H11O7]', 'm/z 244.0542 [C13H10N1O4]', 'm/z 245.0345 []', 'm/z 246.0336 [C12H8N1O5]', 'm/z 247.0144 []', 'm/z 283.0276 []', 'm/z 297.0802 [C17H13O5]', 'm/z 298.081 [C20H12N1O2]', 'm/z 299.0619 [C15H11N2O5]', 'm/z 300.0619 [C19H10N1O3]', 'm/z 301.0539 [C18H9N2O3]', 'm/z 302.0531 []', 'm/z 317.0719 [C15H13N2O6]', 'm/z 355.0672 [C15H15O10]', 'm/z 356.068 [C18H14N1O7]', 'm/z 357.0571 [C18H13O8]', 'm/z 371.0986 [C16H19O10]', 'm/z 372.0995 [C15H18N1O10]', 'm/z 373.0818 []', 'm/z 374.0817 [C18H16N1O8]', 'm/z 375.0764 [C18H15O9]']

**Immediate Reporting of Average Concentration of All Compounds**

**Table S1.** Sample VOC concentration (ppb) reported by VAPOR.

| **Plant Genotype** | **m/z 44.050 [C_2_H_6_N]**  **Ethanamine** | **m/z 47.050 [C_2_H_7_O]**  **Ethanol** | **m/z 59.050 [C_3_H_7_O]**  **Acetone** | **m/z 63.044 [C_2_H_7_O_2_]**  **Ethylene glycol** | **m/z 69.070 [C_5_H_9_]**  **Isoprene** | **m/z 71.050 [C_4_H_7_O]**  **Methacrolein/ Methyl vinyl ketone** |
| --- | --- | --- | --- | --- | --- | --- |
| 103_52-225_R1_PM | 0.016 | 1.438 | 3.095 | 0.708 | 1.014 | 0.253 |
| 100_52-225_R2_PM | 0.059 | 1.471 | 3.944 | 0.884 | 1.627 | 0.343 |
| 34_52-225_R3_PM | 0.105 | 0.259 | 1.700 | 0.752 | 1.396 | 0.265 |
| 98_#1428_R1_PM | 0.155 | 0.670 | 1.073 | 0.423 | 3.978 | 0.171 |
| 18_#1428_R2_PM | 0.199 | 0.325 | 2.893 | 0.790 | 3.820 | 0.387 |
| 14_#1428_R3_PM | 0.245 | 0.122 | 1.001 | 1.437 | 3.309 | 0.320 |
| 10_ILL-101_R1_PM | 0.270 | 0.000 | 1.877 | 1.507 | 3.359 | 0.496 |
| 84_ILL-101_R2_PM | 0.265 | 6.390 | 0.767 | 1.301 | 9.529 | 0.323 |
| 50_ILL-101_R3_PM | 0.299 | 0.000 | 0.160 | 0.706 | 7.957 | 0.184 |
| 66_BESC 24_R1_PM | 0.291 | 2.807 | 1.603 | 0.922 | 9.528 | 0.231 |
| 3_BESC 24_R2_PM | 0.304 | 1.169 | 2.097 | 1.095 | 1.894 | 0.285 |
| 72_BESC 24_R3_PM | 0.037 | 0.000 | 0.000 | 0.560 | 7.434 | 0.119 |
| 9_D-124_R3_PM | 0.159 | 0.000 | 2.488 | 1.874 | 6.686 | 0.711 |
| 13_D-124_R2_PM | 0.180 | 0.000 | 1.242 | 1.684 | 7.939 | 0.490 |
| 89_D-124_R1_PM | 0.198 | 0.000 | 1.514 | 1.987 | 13.958 | 0.657 |
| 4_ILL-101_R1_FS | 0.060 | 0.000 | 0.000 | 0.270 | 10.091 | 0.039 |
| 42_ILL-101_R2_FS | 0.087 | 0.000 | 2.378 | 0.322 | 9.050 | 0.094 |
| 75_ILL-101_R3_FS | 0.134 | 0.000 | 0.000 | 0.315 | 7.247 | 0.026 |
| 108_D-124_R1_FS | 0.170 | 0.000 | 0.000 | 0.133 | 2.449 | 0.000 |
| 70_D-124_R2_FS | 0.222 | 0.000 | 0.000 | 0.398 | 3.847 | 0.128 |
| 26_D-124_R3_FS | 0.259 | 0.000 | 0.000 | 0.579 | 5.172 | 0.078 |
| 94_BESC 24_R3_FS | 0.278 | 0.000 | 0.000 | 0.475 | 7.646 | 0.108 |
| 45_BESC 24_R2_FS | 0.276 | 0.000 | 0.000 | 0.283 | 7.660 | 0.046 |
| 107_BESC 24_R1_FS | 0.278 | 0.000 | 0.000 | 0.121 | 7.391 | 0.000 |
| 25_52-225_R1_FS | 0.297 | 0.000 | 0.000 | 0.235 | 4.075 | 0.000 |
| 57_52-225_R2_FS | 0.299 | 0.000 | 0.000 | 0.263 | 3.564 | 0.000 |
| 78_52-225_R3_FS | 0.161 | 0.000 | 1.323 | 0.000 | 4.600 | 0.014 |
| 54_#1428_R1_FS | 0.225 | 0.000 | 0.000 | 0.234 | 4.629 | 0.174 |
| 7_#1428_R2_FS | 0.300 | 0.000 | 0.000 | 0.180 | 6.064 | 0.075 |
| 29_#1428_R3_FS | 0.386 | 0.000 | 0.000 | 0.203 | 5.915 | 0.217 |

**Ranking of compounds based on prevalence among all VOCs**

**Table S2.** Ranking of VOCs according to frequency of occurrence among the plant samples. Note that the table only reports the top 30 but the measurement listed more than 290 compounds.

| **Rank** | **VOC** | **Frequency** |
| --- | --- | --- |
| 1 | m/z 167.0544 [C5H11O6] Xylonic acid | 1 |
| 2 | m/z 169.0351 [] | 1 |
| 3 | m/z 69.07034 [C5H9] Isoprene | 1 |
| 4 | m/z 70.07196 [] | 0.966667 |
| 5 | m/z 173.1616 [C9H21N2O1] | 0.966667 |
| 6 | m/z 174.1261 [C12H16N1] | 0.966667 |
| 7 | m/z 134.0807 [C5H12N1O3] | 0.966667 |
| 8 | m/z 160.1153 [C11H14N1] | 0.966667 |
| 9 | m/z 116.0703 [C5H10N1O2] | 0.966667 |
| 10 | m/z 88.07581 [C4H10N1O1] | 0.966667 |
| 11 | m/z 159.1124 [C7H15N2O2] | 0.966667 |
| 12 | m/z 114.0541 [C5H8N1O2] | 0.933333 |
| 13 | m/z 187.1424 [C9H19N2O2] | 0.933333 |
| 14 | m/z 133.0786 [C8H9N2] | 0.9 |
| 15 | m/z 170.0358 [] | 0.9 |
| 16 | m/z 188.1451 [C13H18N1] | 0.9 |
| 17 | m/z 68.06071 [] | 0.866667 |
| 18 | m/z 41.03881 [C3H5]  Cyclopropene | 0.866667 |
| 19 | m/z 39.02343 [] | 0.866667 |
| 20 | m/z 130.0857 [C6H12N1O2] | 0.866667 |
| 21 | m/z 174.1674 [] | 0.866667 |
| 22 | m/z 175.0887 [C10H11N2O1] | 0.866667 |
| 23 | m/z 70.02959 [C3H4N1O1] | 0.8 |
| 24 | m/z 90.01776 [C2H4N1O3] | 0.8 |
| 25 | m/z 67.05438 [C5H7] | 0.8 |
| 26 | m/z 158.0906 [] | 0.8 |
| 27 | m/z 88.02164 [] | 0.8 |
| 28 | m/z 116.0201 [] | 0.766667 |
| 29 | m/z 173.0875 [C7H13N2O3] | 0.766667 |
| 30 | m/z 161.1308 [C7H17N2O2] | 0.766667 |

**Sample list of gases with high variance over different plant species** (set model value: 0.1)

['m/z 31.00701 []', 'm/z 31.01847 []', 'm/z 32.21715 []', 'm/z 32.23437 []', 'm/z 32.25822 []', 'm/z 32.99366 []', 'm/z 33.02157 []', 'm/z 33.03373 [C1H5O1]', 'm/z 33.99351 []', 'm/z 37.02731 []', 'm/z 37.03157 []', 'm/z 39.02343 []', 'm/z 39.03345 []', 'm/z 39.61042 []', 'm/z 41.03881 [C3H5]', 'm/z 42.04307 []', 'm/z 43.01844 []', 'm/z 43.05443 [C3H7]', 'm/z 43.68491 []', 'm/z 44.02237 []', 'm/z 44.98113 []', 'm/z 45.03463 [C2H5O1]', 'm/z 46.03188 []', 'm/z 46.06504 [C2H8N1]', 'm/z 47.01331 []', 'm/z 47.04945 [C2H7O1]', 'm/z 48.25941 []', 'm/z 51.04447 []', 'm/z 55.03981 []', 'm/z 55.05458 [C4H7]', 'm/z 57.03384 [C3H5O1]', 'm/z 57.07029 [C4H9]', 'm/z 58.06682 [C3H8N1]', 'm/z 59.04966 [C3H7O1]', 'm/z 60.04716 []', 'm/z 61.02909 [C2H5O2]', 'm/z 63.04421 [C2H7O2]', 'm/z 65.06 []', 'm/z 67.05438 [C5H7]', 'm/z 69.03369 [C4H5O1]', 'm/z 69.07034 [C5H9]', 'm/z 70.07196 []', 'm/z 71.0496 [C4H7O1]', 'm/z 72.0448 [C3H6N1O1]', 'm/z 73.02891 [C3H5O2]', 'm/z 73.06516 [C4H9O1]', 'm/z 74.06035 [C3H8N1O1]', 'm/z 74.09636 []', 'm/z 75.02612 []', 'm/z 75.04438 [C3H7O2]', 'm/z 77.02347 [C2H5O3]', 'm/z 77.05986 [C3H9O2]', 'm/z 79.03914 [C2H7O3]', 'm/z 81.06969 [C6H9]', 'm/z 83.08558 [C6H11]', 'm/z 85.06462 [C5H9O1]', 'm/z 87.04437 [C4H7O2]', 'm/z 87.07253 []', 'm/z 88.02164 []', 'm/z 88.04909 []', 'm/z 88.07581 [C4H10N1O1]', 'm/z 89.02334 [C3H5O3]', 'm/z 89.06186 [C4H9O2]', 'm/z 91.04061 [C3H7O3]', 'm/z 91.06341 []', 'm/z 92.04337 []', 'm/z 93.03758 []', 'm/z 93.06947 [C7H9]', 'm/z 94.03747 []', 'm/z 95.01496 []', 'm/z 95.03305 [C2H7O4]', 'm/z 95.04832 [C6H7O1]', 'm/z 97.10068 [C7H13]', 'm/z 99.07991 [C6H11O1]', 'm/z 101.0594 [C5H9O2]', 'm/z 101.0952 [C6H13O1]', 'm/z 103.0391 [C4H7O3]', 'm/z 103.0783 [C5H11O2]', 'm/z 105.0681 [C3H9N2O2]', 'm/z 107.0682 [C4H11O3]', 'm/z 107.0825 [C3H11N2O2]', 'm/z 109.0499 [C3H9O4]', 'm/z 109.0677 [C7H9O1]', 'm/z 110.9809 []', 'm/z 111.0475 [C6H7O2]', 'm/z 112.0474 []', 'm/z 113.0277 []', 'm/z 114.0541 [C5H8N1O2]', 'm/z 115.0749 [C6H11O2]', 'm/z 115.1108 [C7H15O1]', 'm/z 116.0703 [C5H10N1O2]', 'm/z 117.0747 []', 'm/z 117.1001 [C5H13N2O1]', 'm/z 119.0847 [C9H11]', 'm/z 126.9033 []', 'm/z 129.0905 [C7H13O2]', 'm/z 130.0857 [C6H12N1O2]', 'm/z 133.0786 [C8H9N2]', 'm/z 134.0807 [C5H12N1O3]', 'm/z 136.0219 [C3H6N1O5]', 'm/z 143.1052 [C8H15O2]', 'm/z 145.1321 [C7H17N2O1]', 'm/z 149.0225 [C8H5O3]', 'm/z 159.0621 [C7H11O4]', 'm/z 159.1124 [C7H15N2O2]', 'm/z 160.1153 [C11H14N1]', 'm/z 161.1308 [C7H17N2O2]', 'm/z 163.1116 [C11H15O1]', 'm/z 167.033 [C8H7O4]', 'm/z 167.0544 [C5H11O6]', 'm/z 173.1616 [C9H21N2O1]', 'm/z 174.1261 [C12H16N1]', 'm/z 177.054 [C10H9O3]', 'm/z 187.1424 [C9H19N2O2]', 'm/z 203.9431 []', 'm/z 204.9448 []', 'm/z 223.0551 [C18H7]', 'm/z 223.0942 [C7H15N2O6]', 'm/z 224.0609 []', 'm/z 225.0428 [C10H9O6]', 'm/z 226.0428 []', 'm/z 227.0336 [C13H7O4]', 'm/z 297.0802 [C17H13O5]', 'm/z 299.0619 [C15H11N2O5]', 'm/z 300.0619 [C19H10N1O3]', 'm/z 329.8372 []', 'm/z 330.8476 []', 'm/z 331.849 []']
